# Clinical and Functional Outcomes of Anatomical Plating in Distal Humerus Fractures in Adults

**DOI:** 10.7759/cureus.35581

**Published:** 2023-02-28

**Authors:** Ramavtar Saini, Anshu Sharma, Kuldeep S Rathore, Shehbaz S Sidhu

**Affiliations:** 1 Orthopaedics, Geetanjali Medical College and Hospital, Udaipur, IND

**Keywords:** early mobilization, mayo elbow performance score, anatomical locking plates, anatomical reduction, inrtra-articular fracture, distal end humerus fracture

## Abstract

Background

Overall, 2% of all adult fractures are attributed to fractures of the distal humerus, including both supracondylar and intercondylar fractures. According to recent studies, stable fixation with anatomical reduction of intra-articular fragments and early mobilization is required for best outcomes. This study included patients with distal end humerus fractures treated by open reduction and internal fixation (ORIF) with anatomical locking plates and evaluated their clinical outcomes.

Methodology

This prospective study was conducted at a medical college teaching hospital in southern Rajasthan, India. In total, 20 adult patients with distal end humerus fractures who presented to the orthopedic outpatient department or casualty were admitted. Patients were treated by ORIF with anatomical locking plates, followed up, and evaluated for clinical and functional outcomes.

Results

Using the Mayo Elbow Performance Score, out of 20 cases, excellent results were noted in five patients, good results in seven patients, fair results in six patients, and poor results in two patients.

Conclusions

Locking plates are reliable and effective management options for distal humerus fractures. As locking plates are strong and rigid, the period of immobilization can be decreased. Early mobilization helps in preventing joint stiffness and fixed deformity of the joint.

## Introduction

The elbow is a synovial joint with complex hinges and is vital in positioning the hand in space. In adults, 2% of all fractures and 30% of bony injuries around the elbow are attributed to fractures of the distal humerus [1]. Distal humerus fractures have a bimodal age distribution. With ever-increasing vehicular traffic, there is an increase in road traffic accidents and the concomitant incidence of distal humerus fracture is on the rise, especially among younger individuals. According to a study conducted by Court-Brown and Caesar, increasing life expectancy and osteoporosis prevalence are associated with the increasing number of osteoporotic distal humerus fractures in older individuals [2].

These fractures are commonly caused by axial loading through the joint with the elbow flexed more than 90 degrees. The olecranon here acts as a wedge forcing itself between the two humeral condyles. Hence, most distal humerus fractures in adults are intra-articular and involve both condyles [3].

The Association of the Study of Internal Fixation (AO/ASIF) has defined the following three types of distal humeral fractures [4]: (1) Type A: an extra-articular (supracondylar) fracture; Type B: partial intra-articular (unicondylar) fractures; and Type C: total intra-articular (bicondylar) fractures.

Intra-articular bicondylar fractures (AO Type C) are difficult to manage as malunion, stiffness, and osteoarthritis are common. It was in 1811 when Desault pronounced that these fractures are the most difficult to treat [5]. Management is determined by the pattern of the fracture and the degree of comminution and can be complex and time-consuming. The management of these fractures varies from conservative plaster of Paris cast immobilization (bag of bones) to fully invasive open reduction and internal fixation (ORIF) [6].

Pseudo-arthrosis with gross instability or a painful stiff elbow is the usual outcome of conservative management of these fractures. Moreover, by closed methods, anatomical reduction of the articular surface is not always possible. As a result, many surgeons now advocate open anatomical reduction with stable fixation followed by early mobilization of the elbow to gain acceptable functional results [7].

At present, CT scans with three-dimensional reconstructions of complex articular fractures, a better understanding of locking plate principal, and the availability of pre-contoured periarticular anatomical plates provide support in the management of these fractures.

The ultimate goal is to achieve uncomplicated healing of soft tissue, the union between distal fragment and shaft, diaphyseal bone block restoration, and a stable and mobile elbow joint.

With time, our ability to treat these fractures has improved substantially due to a better understanding of fracture morphology, surgical approaches, and advanced implant design. This study included patients with distal end humerus fractures who were treated by ORIF with anatomical locking plates and evaluated their clinical and functional outcomes.

## Materials and methods

This study was performed at a tertiary care medical college center in southern Rajasthan after obtaining ethics committee clearance (Institutional Ethics Committee, Geetanjali Medical College and Hospital, Udaipur, Rajasthan: GU/HREC/EC/2021/1927). Adult patients of both sexes with displaced fractures of the distal end of the humerus who were medically fit and provided informed consent were included. Both closed and compound cases were included. In compound cases, only those included in Gustilo-Anderson classification Types I and II were included in the study [8]. All patients included in the study were adequately followed and supervised to achieve the best possible results.

Initial management of resuscitation

Patients were screened for injuries to other systems because they are closely associated with high-velocity trauma. Any associated neurovascular deficit was ruled out at this stage and noted. Gentle manipulation was done to achieve the gross reduction of the fracture segment and a support slab was applied. True anteroposterior and lateral radiographs were taken, along with CT scans with 3D reconstruction. On admission, a detailed history of injury including the mode and severity of the injury was obtained.

Preoperative assessment and planning

Any associated systemic injuries were investigated, and a local examination of the part was done to check the skin condition. Before surgery, the fracture characteristics were fully examined on radiographs, including bone quality, fracture pattern, the extent of comminution, and the articular involvement. All relevant laboratory investigations including coagulation studies were performed along with a pre-anesthetic checkup. If the patient’s medical status was in doubt, a medical team consultation was conducted. Patients were counseled about the outcome of the final functional results.

Classification

Fractures were classified according to the Riseborough and Radin classification, which is based on displacement and rotation [9]. The classification was as follows: Type I: minimally displaced intra-articular fracture; Type II: displaced intra-articular fracture, not rotated; Type III: fractures fragments are displaced and rotated; and Type IV: comminuted fracture.

Surgical technique

The posterior olecranon osteotomy approach was used. Fixation devices used included medial and lateral anatomical locking plates, cancellous and cortical screws, tension band wires, and K wires.

Postoperative care and follow-up

Postoperatively, on the third day, the wound was examined. Patients were discharged on the fifth to seventh postoperative day, and antibiotics and analgesics were given as per requirement. On the 12th to 14th postoperative day, the sutures were removed. After the third week, patients were instructed to perform elbow and wrist range of motion (ROM) exercises, both active and passive, as tolerated, three to four times daily. They were also advised to use an arm sling pouch during the night. It was recommended that they avoid lifting heavy objects or exerting the affected upper limb. Regular follow-up was done in the sixth week, the third month, and then every three months up to one year. A thorough clinical and radiological examination was performed at the follow-up appointment. The Mayo elbow performance score (MEPS) was used to assess the functional outcomes of patients [10].

## Results

This study included a total of 20 adults with distal end humerus fractures, extending into the intercondylar region treated with ORIF using anatomical locking plates.

The youngest patient was 20 years old while the oldest was 65 years old. The average age was 39.05 years. Most patients in our study were between the ages of 31 and 40, which is the young working-age group. As males are more active in outdoor activities, there was a higher incidence in males. The male-to-female ratio in our study was 3:2. In our study, 13 (65%) patients had right-side involvement and seven (35%) had left-side involvement.

The most prevalent type of injury encountered in this study was road traffic accidents in 12 cases, a fall in six cases, and assault in two cases (Table 1).

**Table 1:** Distribution of the cases according to the mode of the injury.

Mode of injury	Number	Percentage (%)
Assault	02	10%
Road traffic accidents	12	60%
Fall	06	30%

Fractures were classified using the Riseborough and Radin classification. Out of the 20 cases studied, nine (45%) had Type III fractures, while 11 (55%) cases had Type IV fractures. In our study, one (5%) case had a history of massage of the elbow by a quack, while 19 (95%) cases had no such history.

In our study, the duration between trauma and surgery varied. In some cases, patient ignorance was the cause, while in others medical fitness for surgery delayed the definitive surgical process. The majority of the patients (16 cases) were operated on between 24 hours to one week. Overall, 13 (65%) patients were operated on under regional anesthesia (supraclavicular block), while seven (35%) cases were operated on under general anesthesia.

The minimum follow-up was six months. Most patients, 17 (85%), were followed up for six to nine months, with an average follow-up of 8.5 months. In our study, eight (40%) patients had arcs of motion in the range of 81 to 100 degrees while 12 (60.00%) patients had an arc of more than 100 degrees (Table 2).

**Table 2:** Distribution of the cases according to extension loss.

Extension loss of the elbow (degree)	Number	Percentage (%)
0–10	6	25%
11–20	11	60%
21–30	3	15%
Total	20	100%

In our study, the most common complication was the stiffness of the elbow joint seen in three (15%) cases, followed by hardware pain in two (10%) cases.

At the final follow-up, in our study, five (25%) patients had excellent scores, seven (35%) had good scores, six (30%) had fair scores, and two (10%) patients had poor scores (Table 3).

**Table 3:** Distribution of the cases according to the Mayo elbow performance score.

Mayo elbow score	Number	Percentage (%)
Excellent	05	25%
Good	07	35%
Fair	06	30%
Poor	02	10%
Total	20	100%

In total, 18 (90%) patients returned to their old profession after a fracture union while two (10%) had to change their profession.

**Figure 1:**
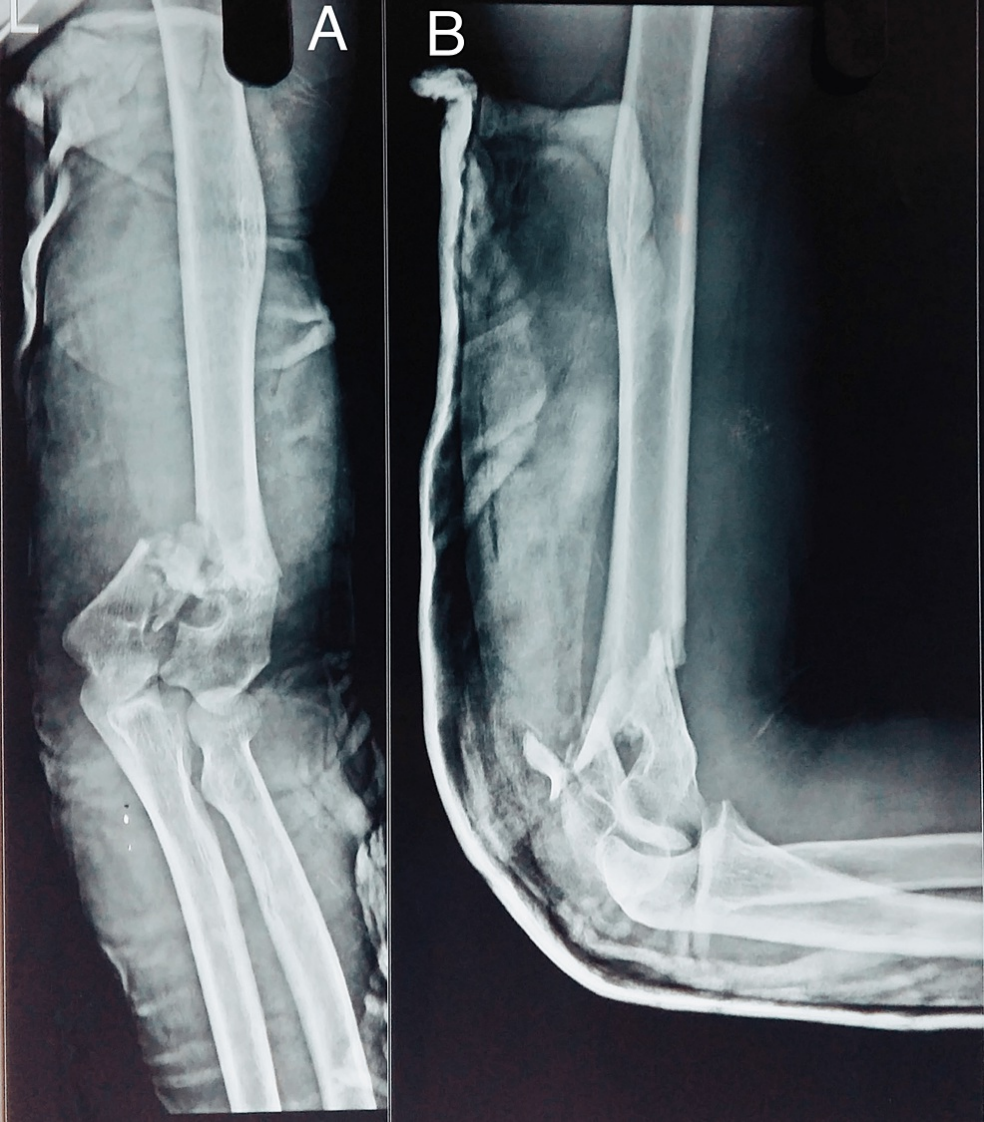
Preoperative radiograph. Preoperative radiograph showing comminuted intra-articular fracture of the distal humerus. A: anteroposterior view; B: lateral view.

**Figure 2:**
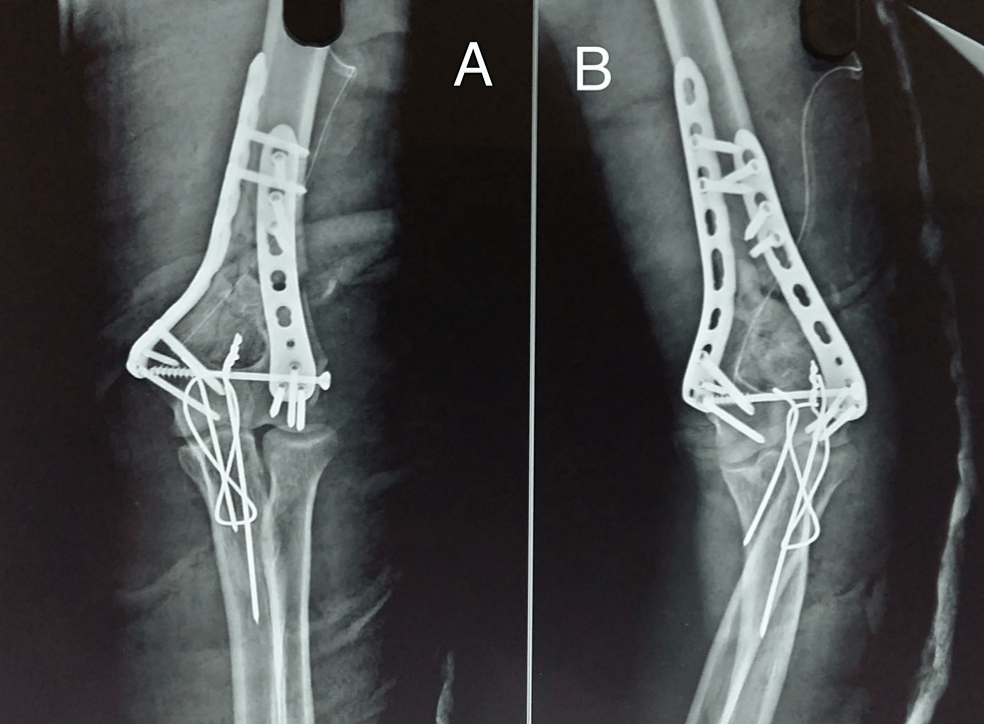
Postoperative radiograph. Immediate postoperative radiograph showing fixation of the fracture with distal humerus anatomical locking plates using the posterior olecranon osteotomy approach. A: anteroposterior view; B: lateral view.

**Figure 3:**
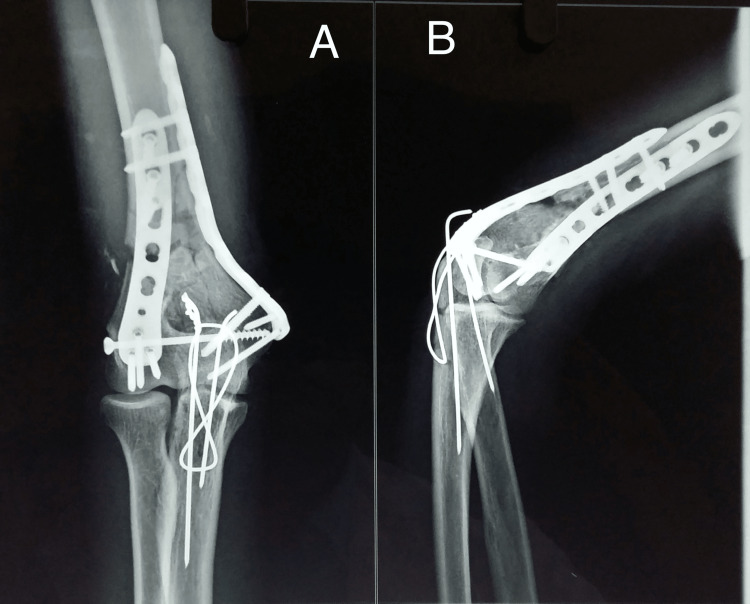
Three-month postoperative follow-up radiograph. Third-month postoperative follow-up radiograph showing the progressive union of the fracture and olecranon osteotomy. A: anteroposterior view; B: lateral view.

**Figure 4:**
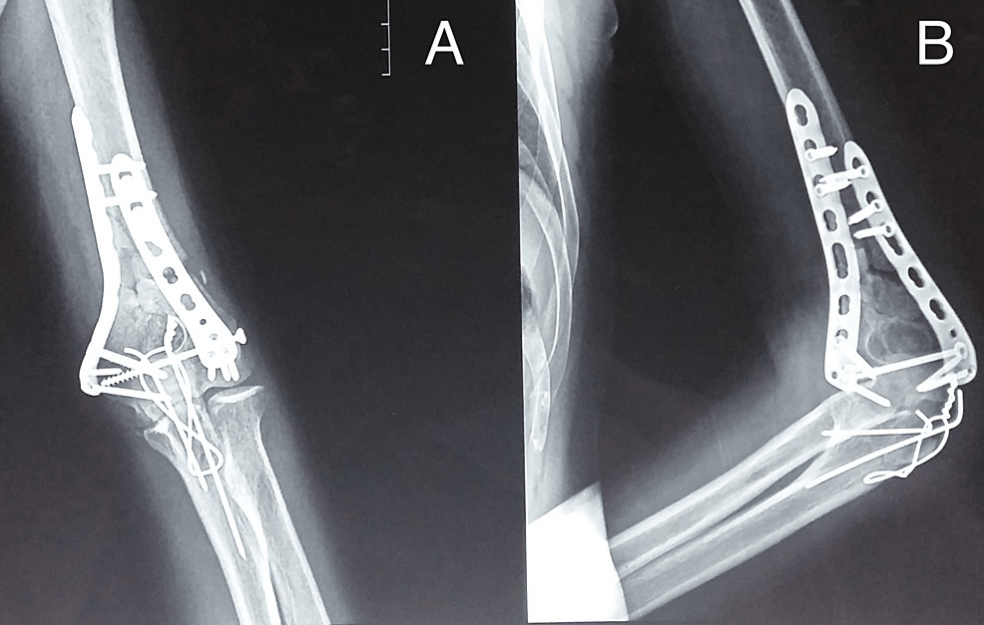
Sixth-month postoperative follow-up radiograph. The final follow-up radiograph showing fracture union. A: anteroposterior view; B: lateral view.

**Figure 5:**
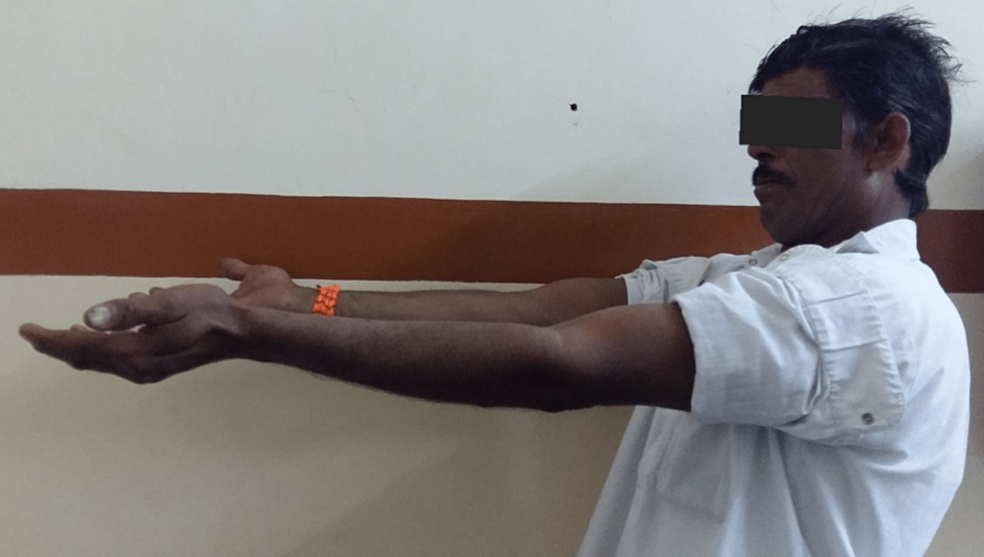
Clinical photograph showing extension movement at the elbow. Clinical photograph at the final sixth-month postoperative follow-up showing good extension range of movement at the elbow joint.

**Figure 6:**
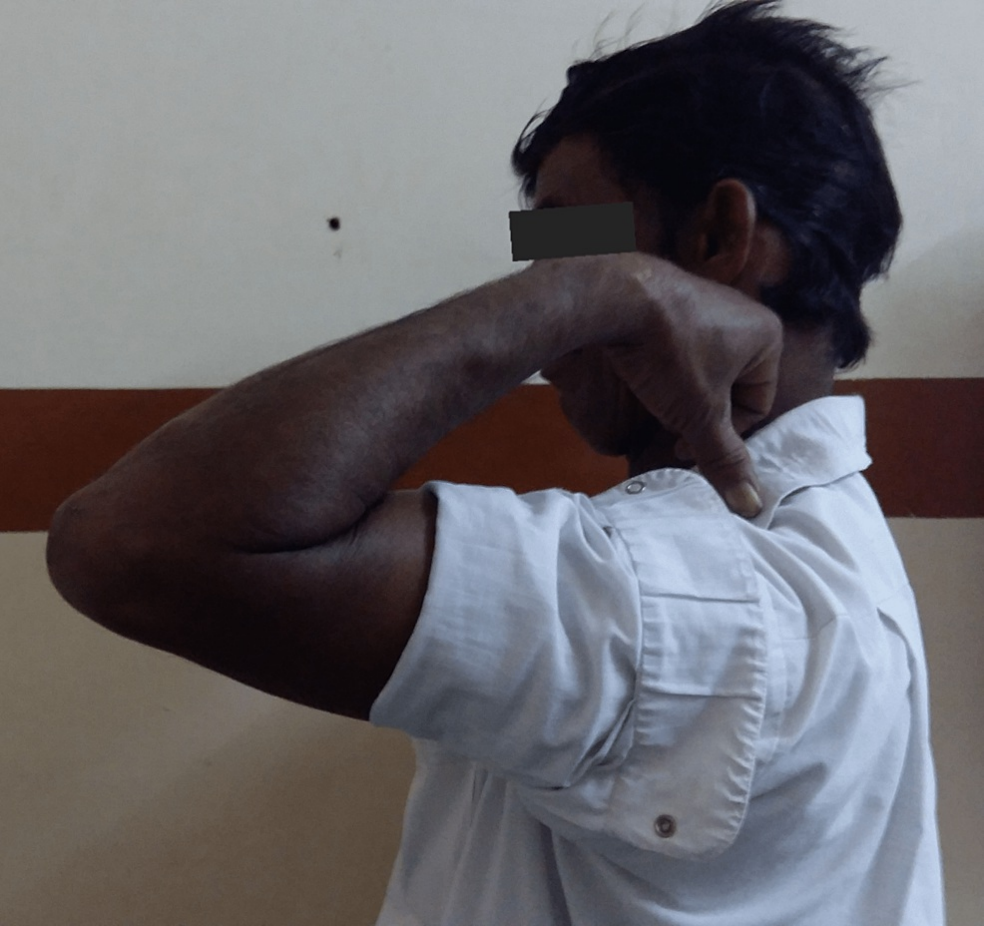
Clinical photograph showing flexion movement at the elbow. Clinical photograph at the final sixth-month postoperative follow-up showing good flexion range of movement at the elbow joint.

The radiographic images of one of our study patients are shown in Figures 1-4 and clinical images at the final follow-up are shown in Figure 5 and Figure 6.

## Discussion

Because of the nature of the injury, distal humerus fractures in adults are difficult to treat. The conservative treatment approach to these fractures cannot ensure good reconstruction of the articular surface or allow for the early mobilization of the elbow, both of which are crucial in achieving good function. With the development of newer, stronger biocompatible materials, a wider range of hardware options is now available for the reduction and fixation of distal humerus fractures. Anatomically pre-contoured plates enable the surgeon to maintain the original articular congruence required to prevent post-traumatic arthritis, allowing for a more rapid and progressive rehabilitation.

In our series of 20 cases, the maximum number of cases was in their fourth decade. The series by Cassebaum et al. [11] showed a shift towards the fifth and sixth decades. We had a male-to-female ratio of 3:2 which is similar to that reported by Miller et al. [12] and Mckee et al. [13]. In our study, the most common mode of injury was road traffic accidents in 12 (60%) cases, which is comparable to studies reported by Henley [14] and McKee et al. [15].

All 20 cases were classified in accordance with the Riseborough and Radin classification depending on the fracture pattern and extent of comminution. This classification has some therapeutic significance and provides some guidance to management and prognosis. All patients had Type III and Type IV fractures. No patients had Type I and Type II fractures as they were managed conservatively.

The surgeon prefers the lateral position with the patient’s arm hanging by the side. Furthermore, flexion of the elbow in this position provides a clear view of the articular surface of the distal humerus. We agree with Jupiter and Mehne [3] that the trans-olecranon approach provides excellent exposure for articular surface reconstruction, particularly in AO Type C3 fractures.

Complex distal humeral fractures are not amenable to single-column plating systems. Fixation with double plating is currently recommended based on clinical and biomechanical studies. In our study, dual-column fixation using anatomically pre-contoured locking plates and cancellous screws was done in all cases which provided the most secure fixation.

Regarding early mobilization, several authors, including Jupiter and Mehne [3] and Lansinger and Måre [16], have mentioned mobilization as early as the pain subsides with intermittent use of splints and braces. In our study, we mobilized patients between two to three weeks postoperatively depending on patient compliance.

As the primary complication of this injury is decreased movement of the elbow, the ROM was noted in each patient during the follow-up period. At the final follow-up, 11 (40%) cases achieved an arc of motion of more than 100 degrees, seven (43.33%) had an arc of 81-100 degrees, and two (13.33%) had a range of 61-80 degrees. Our findings were comparable with the studies conducted by Henley [14], Mckee et al. [15], and Jupiter and Mehne [3].

A review of the literature on internal fixation of these fractures revealed a relatively high incidence of ulnar nerve neuropraxia [17], but no ulnar nerve palsy was observed in our study. However, we agree with Ring and Jupiter [18] that whenever a peroperative assessment reveals a compromised ulnar nerve, it should be transposed anteriorly.

In our study, the most common complication was the stiffness of the elbow joint seen in three (15%) cases, followed by hardware pain in two (10%) cases. After treatment of such a dreaded injury, one should accept some limitations of movements at the elbow. Some causes of impaired joint function are discussed below.

The first is the mechanical incongruity of the joint from a bone block, a malunited trochlea, and obliteration of the olecranon fossa, and destruction of articular cartilage or other bony injuries to components of the joint.

The second type of impaired function also appeared to be from excessive periarticular fibrosis as a result of the original trauma, faulty surgical technique, excessive mobilization or thickening of the capsule, and shortening of collateral ligaments.

A third factor that contributed to both mechanical incongruity and periarticular fibrosis was an inadequate fixation of the fragments.

In all reports, there has been a great variation [19,20]. In our study, six (25%) patients had an extension loss of 0-10 degrees, eleven (60%) patients had an extension loss of 11-20 degrees, and three (15%) patients had an extension loss of 21-30 degrees [19,20].

The MEPS was used in this study for the final outcome as it takes into consideration additional features such as pain and the activity level with the ROM at the elbow. We had five cases with excellent results, seven with good results, and six patients with fair results. Unfortunately, two patients had poor results, which was mainly due to non-compliance to the physiotherapy regimen and late presentation with a history of massage. Our findings reinforce the desirability of an operative approach to these fractures and compare favorably with the literature [21].

The age group in the present series was relatively younger, with good bone stock, and this may have been the reason for a lack of fixation failure and the higher percentage of acceptable results. Holdsworth and Mossad also indicated that old age is no contraindication for surgical management of these fractures. and the final outcomes are more dependent on the quality of the bone rather than the chronological age of the patient [22].

We agree with Patel et al. that ORIF with the pre-contoured distal humerus anatomical locking plate system is a good treatment method for complex supra and intercondylar fracture of the distal humerus to achieve restoration of the articular surface anatomy, stable fixation, and early mobilization with good functional outcomes and low rates of complications [23].

In studies reported by Greiner et al. [19] and Korner et al. [24], in ORIF of these complicated distal humerus fractures with anatomical plate, there is an association between delayed union after olecranon osteotomy and ulnar nerve irritation; however, in our study, no such complications were recorded [19,24].

According to a study by Kumbaraci et al. [25], the maximum percentage of distal humerus fracture patients treated with pre-contoured anatomical plates had to change their profession from their preinjury work; however, in our study, only two (10%) patients had to change their profession.

Our study did have certain limitations. First, this study had a small sample size of 20 patients. Second, this was a single-center study.

## Conclusions

Within the limitations of the present study, we are able to form the practical concept that every case of fracture of the intra-articular humerus should draw the attention of a surgeon with a wide range of thoughts. Balanced assessment should be performed regarding the need of the patient according to age and occupation, type and extent of the injury, and the best possible treatment modality based on fracture characteristics. The key to successful management of intra-articular distal humerus fracture is careful preoperative planning, minimal soft-tissue damage, ulnar nerve exploration, anatomical reduction with rigid dual plate fixation, and early postoperative mobilization.
